# The Inner and Outer Qualities of Extracellular Vesicles for Translational Purposes in Breast Cancer

**DOI:** 10.3389/fimmu.2018.00584

**Published:** 2018-03-26

**Authors:** Esther Schwich, Vera Rebmann

**Affiliations:** Institute for Transfusion Medicine, University Hospital Essen, University Duisburg-Essen, Essen, Germany

**Keywords:** breast cancer, biomarker, liquid biopsy, extracellular vesicles (EV), exosomes, micro-RNA, HLA-G, HER-2/neu

## Abstract

Breast cancer (BC) is the second most common cause of cancer mortality of women worldwide. BC is a systemic disease with a highly heterogeneous course of disease. Therefore, prognostic and diagnostic biomarkers are required to improve the clinical risk management. Cancer-derived or cancer-associated extracellular vesicles (EVs) procured from the bloodstream of BC patients offer a novel platform for the qualitative and quantitative screening and establishment of biomarkers. Here, we focus on common aspects of EVs, on the function of BC-derived EVs and their translational potential considering the EV abundancy, intravesicular as well as outer membrane-anchored composition and current challenges of implementation in clinical practice.

## Introduction: Breast Cancer (BC) Prognosis and Diagnosis

Breast cancer is the second most common cause of cancer mortality of women worldwide and the most prevalent type of cancer among woman. It is a highly heterogeneous disease and subclassified according to the status of the hormone receptors progesterone (PR) and estrogen (ER), overexpression/amplification of the human epidermal growth factor receptor 2 (HER-2), and the basal-like mostly triple-negative (PR−/ER−/HER-2−) subtypes (TNBC) (1). PR and ER status give information about the responsiveness to adjuvant hormonal therapy (2), whereas HER-2/neu amplification is associated with tumor aggressiveness and chemoresistance (3). TNBC subtypes have the worst overall and disease-free survival compared with other subtypes (4), whereas non-metastatic BC is associated with a long-term survival (5, 6). Conventional screening methods encompassing mammography and tissue biopsy investigations and the consequential multimodality therapy approaches including surgery, chemo-, radiation-, and hormone therapy, and antigen-targeted treatments with monoclonal antibodies, have definitely improved BC survival of low-grade and endocrine-responsive tumors (7). For these BC subtypes, the timeline of early detection is less critical than for high-grade or highly proliferating BC subtypes. Although the BC classification is informative, further molecular markers considering alterations in systemic proteomic, glycoproteomic, immune, and nucleic acid profiles, generally observed in BC and other solid malignancies (8) should be established for a better personalized risk management. Ideally, biomarkers should allow refining (i) early detection of BC, (ii) early prediction of relapse, and (iii) chemotherapy response. Heterogeneity of BC evolution within BC patients with tumor spread often leads to mixed clinical responses. Here a molecular profiling of metastatic tumors and a monitoring of metastatic disease response would help to overcome tissue-based diagnostics, especially considering that BC metastasis are commonly localized at relatively inaccessible anatomic sites, such as bone, liver, and lung. Thus, liquid biopsy, procured from the bloodstream, offers as a non-invasive screening source a platform for the qualitative and quantitative detection of cancer-derived or cancer-associated entities, e.g., extracellular vesicles (EVs), circulating tumor DNA (ctDNA), or circulating tumor cells (CTCs). Principally, the genetic information and molecular composition of entities acquired by liquid biopsy should be equal to tissue biopsy, though complications of the latter are avoided (9, 10). In addition, the repeated sampling permits the monitoring of clonal dynamics throughout the course of therapy and early identification of therapeutic resistance drivers or early relapse (9). Analysis of EVs encompasses several advantages over CTCs and ctDNA due to their higher abundancies and stability in the bloodstream, and their functionality in supporting tumor-host cross talk or tumorigenesis in BC. Here, we focus on common aspects of EVs, on the function of BC-derived EVs, their translational potential considering abundancy, intravesicular as well as outer membrane-anchored composition, and challenges of implementation in clinical practice.

## The Status Quo of Common Aspects of EVs

### The Four Categories of EVs

Extracellular vesicles are bi-lipid membrane vesicles secreted by a broad range of cells including tumor cells. The cell of origin controls EV assembly making them highly heterogeneous in size, membrane composition, and molecular content of protein and genetic information (11). According to their size and biogenesis, EVs can be classified in several subtypes: small EVs designated as exosomes (70–150 nm) are secreted *via* a multivesicular-body endocytic process (12); microvesicles (100–1,000 nm) are formed by outward budding and scission of the plasma membrane (13); apoptotic bodies (>500 nm) are generated from plasma membrane blebs of apoptotic tumor cells (14) and oncosomes are non-apoptotic membrane blebs of amoeboid cancerous cells (>1,000–10,000 nm) (15). Hitherto, discrimination relies primarily on their size as the exclusive identification of a specific subtype is virtually impossible by certain invariant housekeeping markers.

### The Outer Membrane-Anchored and Intravesicular Composition of EVs

The outer membrane-anchored composition includes transmembrane or lipid-bound extracellular proteins. The intravesicular or rather the inner content encompasses proteins, lipids, metabolites, and nucleic acids as DNA, mRNA, microRNA, and other non-coding RNAs. Both compositions reflect their parent cell and their activating/health status at time point of generation (16, 17). In general, the lipid membrane of EVs prevents their cargo from enzyme degradation, preserves their functionality and facilitates their transfer even over a long distance (18, 19). Current characterization and verification of EVs is based on the detection of typical EV markers including (i) transmembrane or lipid-bound extracellular proteins, e.g., tetraspanins, cell adhesion molecules, integrins (20–22) and (ii) cytosolic proteins with membrane- or receptor-binding capacity as members of endosomal sorting complexes required for transport of EVs as the tumor susceptibility gene 101 (20, 23). According to recommendation of the international society for EVs (20) EV preparations should be semiquantified for at least one EV-enriched protein of each group mentioned earlier and one appropriate negative control, being an intracellular protein not associated with the plasma membrane or endosomes.

### The Functional Implication of EVs

Extracellular vesicles can exert their functions *via* three mechanisms: (i) receptor–ligand interaction, (ii) direct fusion with plasma membrane, and (iii) internalization of EVs from target cells by phagocytosis, clathrin- and caveolin-mediated endocytosis or micropinocytosis, to transfer genetic information/bioactive molecules to target cells or to participate in intracellular signaling (24). Horizontal transfer of genetic information allows EVs to regulate the recipient cell at a posttranscriptional level, retaining features of the originating tissue or of its microenvironment (11). Depending on their composition, EVs can orchestrate multiple systemic processes such as cell-to-cell communication, and participate in the maintenance of normal physiology (25, 26). Furthermore, EVs can induce gene expression modifications, and activate or suppress immunological responses introducing homeostasis of immune tolerance (27–29). In tumorigenesis, EVs can promote tumor progression by inducing normal cell transformation, remodel the surrounding parenchymal tissue, and modulate the immune system (25, 26, 28). Interestingly, EVs can mediate radiation-induced bystander signaling transferring radiation effects to non-targeted cells (30, 31), and composition of EVs can be modified upon environmental stress such as radiation (32, 33).

### The Challenge to Isolate Pure EV Fractions

Extracellular vesicle purification bears challenges due to their small size and physicochemical properties (34). So far, the choice of purification technique clearly depends on the scientific issue being addressed and on further downstream applications used. Methods for purification encompass precipitation kits, size-exclusion chromatography, and sequential centrifugation followed by an ultracentrifugation step, with the latter being the current gold standard in the field (35, 36). Disadvantages such as reproducibility, potential vesicular disruption impairing the functionality of EVs, or contamination with non-vesicular components, impede the establishment of a standardized method (37). A novel method based on a commercially available bind-elute size-exclusion chromatography might revolutionize EV purification (37).

## The Status Quo of EVs in BC

Extracellular vesicles resemble cancer-derived characteristics and a plethora of proteins is often enriched compared with their cell of origin (38). Hence, recent cancer research is focusing on defining EV subpopulations based on their cargo as an enrichment of a specific cargo in EVs promotes a wide-range of cellular functions in the context of malignancies.

### The Functional Implication of EVs Derived From BC Cell Lines

Different BC cell lines secrete EVs with distinct protein signatures in quantities correlating with increasing metastatic potential, likely facilitating cell migration and metastasization. EVs from murine BC cell lines with metastatic features harbor a distinct set of membrane-anchored proteins including Ceruloplasmin and Metadherin which promote cancer metastasis (39). A potential mediator of BC cell activity, motility, and metastasis seems to be exosomal CD81 secreted from human fibroblasts triggering activation of the autocrine Wnt-planar cell polarity signaling pathway (40). Accordantly, mesenchymal stem-cell-derived EVs promote migration of MCF-7 BC cells by activation of the Wnt-signaling pathway (41). Furthermore, EVs released by human BC cell lines containing the epidermal growth factor receptor (EGFR) ligand amphiregulin increase invasiveness of recipient cancer cells (42). Interestingly, EVs carrying the apoptosis inhibitor Survivin—a protein overexpressed in BC tissues and associated with chemo- and radiotherapy resistance—are linked to tumor recurrence and reduced patient’s survival. The TNBC cell line MDA-MB-231 releases elevated numbers of Survivin-rich EVs upon chemotherapy, thereby promoting tumor survival (43). Furthermore, glutathione S-transferase P1-containing EVs derived from chemoresistant cells seem to induce a drug-resistant phenotype (44). Consistently, therapeutic-induced senescent TNBC cells release enhanced levels of EVs containing key factors linked to cell proliferation, ATP depletion, apoptosis, and the senescence-associated secretory phenotype (45). EVs secreted by tumor-associated macrophages (TAM) can promote BC invasion and metastasis formation, whereas BC-EVs carrying miR-16 inhibit TAM infiltration and polarization of the tumor-supportive M2 macrophage phenotype (46, 47). In addition, compared with EVs from non-tumorigenic cells, miRNA are enriched in BC-EVs, and these EVs can actively convert pre-miRNA into mature miRNA (48). Cells of the non-malignant mammary epithelial cell line HMLE transform into tumorigenic cells upon exosomal uptake of MDA-MB-231-derived miR-10b. Strikingly, EVs can contain double-stranded DNA which represents the entire genome mirroring the mutational status of the parental tumor cell (49, 50). Together, these and other studies (51–53) provide the concept of EVs being a central player in the pathogenesis of BC. Due to the miscellaneous decisive roles of EVs in tumor-promoting processes great efforts have been made to investigate its translational potential as a circulating biomarker in blood of BC patients.

### The Translational Potential of Circulating Blood EV Counts in BC

Likely due to the extracellular acidity of malignant tumors, BC and other tumor entities produce EVs in relative high abundance compared with normal cells which can be locally restricted or systemically released (24, 54–57). Hence, EV count may serve as a surrogate marker for disease detection, whereas EV biochemistry may provide molecular markers to assess tumor severity (58). Systemically released EVs are detectable in nearly all body fluids including blood and ascites fluids, or pleural effusions (59). In primary, non-metastatic, locally advanced BC patients undergoing neoadjuvant chemotherapy (NACT) EV counts are associated with nodal status before NACT suggesting that tumor cells resident in lymph nodes release enhanced EV levels into circulation (54). In addition, EV counts strongly correlate with tumor size before NACT (54). As enhanced EV count before NACT is associated with therapy failure, it is likely that high amounts of EVs negatively impact therapy response. Post NACT high EV levels are associated with a reduced 3-year progression-free and overall survival (54). Interestingly, high EV levels are inversely associated with presence of CTCs (54). Here, it can be hypothesized that CTCs consume EVs for maintaining their BC phenotype in the periphery. Thus, EVs and CTCs isolated from one patient at the same time point uncover different, but yet complementary information on BC disease status and prognosis (60). Consequently, EVs and CTCs should be analyzed simultaneously from liquid biopsies to evaluate minimal residual disease and to improve the understanding of the underlying biology of BC heterogeneity (54).

### The Translational Potential of Intravesicular Components in Circulating Blood EVs of BC

Expression of the cancer marker focal adhesion kinase is significantly elevated in BC-EVs in ascending order with disease stage (61). Similar, levels of carcinoembryonic antigen and cancer antigen 15-3 in circulating EVs of BC patients are linked to cancer progression (62), but do not facilitate a marker of early stage (63). In addition, the proapoptotic splice variant Survivin-2B packaged into circulating serum EVs is discussed as an early diagnostic and/or prognostic marker in BC (64). HSP72 present in EVs from breast and other solid cancers interacts with the toll-like receptor 2 on myeloid-derived suppressor cells which induces their activation and thus promotes an immunosuppressive pathway involved in tumor-induced tolerance (65). Besides intravesicular proteins, a plethora of microRNAs involved in BC progression has been identified in BC-derived EVs (62, 66). Of note, serum-derived EVs of BC patients can contain (i) the RNA-induced silencing complex-loading complex proteins, (ii) the enzyme Dicer, (iii) the transactivating response RNA binding protein (TRBP), and (iv) argonaute 2, which are essential compounds required for miRNA biogenesis. Thus, blood EVs have the potential capacity to convert pre-miRNAs to mature miRNAs (48). Indeed, levels of miR-21 and miR-1246 are elevated in plasma-EVs from BC patients (67) than in healthy controls. Moreover, higher levels of miR-105 in BC serum EVs are associated with metastasis formation representing a potential marker for advanced BC and prognostic outcome during course of disease (68). In serum of BC patients, vesicular, but not cell-free circulating, miR-101, miR-372, and miR-373 are increased compared with healthy controls, the latter being suggested to be indicative for TNBC phenotype (69).

### The Translational Potential of Outer Membrane-Anchored Components in Circulating Blood EVs of BC Patients

Besides the vesicular packaging of cancer cell-specific cargoes, diverse cancer markers are increased on the outer surface of EVs derived from BC patients compared with healthy controls. Expression of the oncogenic marker CD24 on EVs might be clinically relevant in BC and ovarian carcinoma (70). In addition, expression levels of EGFR are increased in BC-EVs in a disease stage dependent manner (61). Not only the tumor-derived molecules MUC1, EGFR, and EpCAM but also the matrix metalloproteinase inducer EMMPRIN are identified in blood-derived EVs of patients with BC and other solid tumor entities (38). Presence of these molecules was significantly associated with a reduced overall survival. Furthermore, the transient receptor potential channel (TRCP5) integrated in the membrane of EVs can mediate chemoresistance to chemo-sensitive BC cells. Indeed, levels of BC plasma-derived EVs carrying TRCP5 correlated with its expression levels in BC tissues and with tumor response to chemotherapy (71). Interestingly, chemotherapy can increase the CD144 or CD62e EV subpopulation, which might be indicative for chemotherapy-related thrombogenicity or vascular damage (72). Glypican-1 (GPC1) is a membrane-anchored protein overexpressed in BC and pancreatic cancer (73) modulating mitogenic effects of various heparin-binding growth factors in these tumors (74, 75). Consequently, presence of GPC1-EVs in the blood of these patients is discussed as promising biomarker (73). Several additional cancer markers have been identified on BC-EVs including HER-2 and HLA-G, both being associated with tumor proliferation, invasiveness, drug resistance, and metastasis formation (76, 77). HER-2 serves as a prognostic indicator for tumor aggressiveness and chemoresistance. EVs derived from HER-2-overexpressing BC cells have been suggested to contribute to this, as they express active HER-2 which potentially binds to the HER-2 antibody, thereby impairing therapy outcome (76). Moreover, resistance to HER-2-targeted therapy seems to be associated with increased levels of TGFβ1 levels in blood EVs derived from HER-2+ BC patients (78). HLA-G, which induces immune tolerance and mediates tumor escape (79, 80), is a biomarker for malignancies comparable to other immune checkpoint molecules (77, 81). High levels of HLA-G in EV fractions positively correlate with disease progression of primary, non-metastatic, locally advanced BC patients undergoing NACT (54). In addition, presence of stem-cell like CTCs is positively associated with high HLA-G levels in EV fractions (54).

## The Challenge to Establish and to Integrate EV-Derived BC Biomarkers in the Clinic

Hitherto, studies on circulating blood EVs dealing with EV counts and phenotypes in BC patients clearly demonstrated the translational potential. However, isolation and characterization methods and corresponding analysis instrumentation limit the translational power of EV research. Instrumentation used for determination of shape, size, and number include electron microscopy, nanoparticle tracking analysis, dynamic light scatter, and resistive pulse sensing. Although the three latter are suitable for high-throughput analyses, these techniques fail to distinguish BC-derived/associated EVs from the total EV population in the blood. Selective identification of discrete sets of EVs can only be achieved *via* BC-derived/associated molecules expressed on the outer EV membrane. The issue is that due to the small diameter size, the vast majority of EVs present only 10 copies of a protein, whereas cells express thousand copies (82). Thus, sensitivity of common flow cytometers reaches their limitation to detect EV populations (83), albeit labeling these few proteins with antibodies conjugated with bright fluorescence dyes. In addition, as multiple small vesicles are simultaneously illuminated as a swarm, EV count within a distinct population is diminished (84, 85). Nevertheless, as accurate determination of EV-derived biomarkers and simple test performing are prerequisite for the successful integration into daily clinical practice, flow cytometric methods appear to be the best choice due to their high throughput and multiplexed capabilities. New methods such as flow cytometric scatter ratio (86) are promising approaches to overcome these difficulties.

A second issue is related to the design of clinical studies and how to define disease markers in BC. By comparing circulating blood EVs in BC patients at diagnosis, pre- and posttreatment, during follow-up, and correlating with clinical and pathologic development, we might be able to predict therapeutic response and patient prognosis. For the establishment of reliable early prediction markers in BC, it is inevitable to analyze blood-derived EVs before tumor diagnosis. This implies a long-term observational study of continuous blood sampling enrolling women undergoing mammography with initial negative test results until tumor diagnosis. This study design, however, is only feasible in national study centers.

## Conclusion and Perspective

At present, clinical studies clearly highlight the translational potential of blood-derived EVs in BC. In future, enumeration and qualitative/qualitative evaluation of EVs, expressing a distinct set of tumor-derived/associated markers, will provide crucial information for the risk management of BC patients in conjunction with their physical examination. Due to the fact that different tumor entities share common phenotypes considering hypoxia, nutrient supply and extracellular acidity, it is likely that a set of certain EV subpopulations of BC patients are meaningful in the integration of risk management protocols for other malignancies. To guarantee a fast implementation of the translational power of blood EVs in clinical practice, it is essential (i) to establish innovative methods for EV isolation and characterization, (ii) to design and conduct clinical discovery and validation studies permitting the monitoring of clonal dynamics of BC and other solid malignancies throughout the course of therapy, and (iii) to establish long-term observational control cohorts. The latter one can best be realized for BC as woman undergo serial preventive medical examination by mammography. The translational power of EVs is not restricted to risk management of cancer patients regarding early identification of tumor development, therapeutic resistance drivers, or early relapse. Introduction of therapeutically engineered endogenous EVs represents promising novel strategies for the efficient and targeted delivery of therapeutics which reduce the cytotoxic side effects of current cancer treatments (87–89).

## Author Contributions

ES and VR: wrote the initial draft and read and approved the final article.

## Conflict of Interest Statement

The authors declare that the research was conducted in the absence of any commercial or financial relationships that could be construed as a potential conflict of interest.
